# Gewalt gegen Rettungsdienstpersonal

**DOI:** 10.1007/s00103-022-03564-5

**Published:** 2022-07-21

**Authors:** Fredericke Leuschner, Anne T. Herr, Paulina Lutz, Lena Fecher, Michaela Selzer

**Affiliations:** 1 Kriminologische Zentralstelle e. V., Luisenstraße 7, 65185 Wiesbaden, Deutschland; 2 Hessische Hochschule für öffentliches Management und Sicherheit, Wiesbaden, Deutschland; 3 Bayerisches Rotes Kreuz, München, Deutschland

**Keywords:** Angriffe, Folgen von Gewalt, Viktimisierung, Deeskalation, Aus- und Fortbildung, Attacks, Consequences of violence, Victimisation, De-escalation, Education/training

## Abstract

**Hintergrund und Ziel:**

Angriffe gegen Rettungsdienstpersonal werden zwar medial und politisch vermehrt diskutiert, was sich auch in politischen Initiativen und Gesetzesänderungen widerspiegelt, die wissenschaftliche Studienlage in Deutschland ist allerdings noch lückenhaft und zeichnet insbesondere hinsichtlich der Prävalenzen solcher Vorfälle kein einheitliches Bild. Der vorliegende Artikel widmet sich einerseits den Prävalenzen, andererseits werden situative Eskalationsfaktoren sowie Folgen der Vorfälle und Wünsche der Einsatzkräfte in Bezug auf Angriffe dargelegt.

**Methoden:**

Mittels eines Mixed-Methods-Ansatzes, der eine Langzeitbefragung zu den Häufigkeiten von Gewaltdelikten in Form eines Onlinefragebogens und qualitative Interviews mit Expert*innen und Betroffenen umfasst, wurde Rettungsdienstpersonal im Zeitraum von Mai bis August 2021 befragt.

**Ergebnisse:**

Es zeigt sich, dass Angriffe, insbesondere verbaler Art, zum Arbeitsalltag von Rettungsdienstpersonal gehören. Im Durchschnitt wurden wöchentlich 29 % der Befragten beleidigt, belästigt oder verbal bedroht. Aber auch körperlichen Angriffen waren pro Woche durchschnittlich 8 % der Befragten ausgesetzt. Gleichzeitig wird hinsichtlich der Nachbetreuung und bezüglich der Ausbildungslage der Wunsch nach Anpassungen und Änderungen betont.

**Diskussion:**

Eine Ausbildung sowie Schulungen, die für Gefahren sensibilisieren, Deeskalationsansätze in den Blick nehmen und Eigensicherung thematisieren, könnten das Risiko von Angriffen und somit Belastungen dieser Art im Berufsalltag senken.

## Abstract (en)

**Background:**

Attacks against emergency medical services are increasingly discussed in the media and in politics, which is reflected in political initiatives and legislative changes. However, there is a rather low number of scientific studies on this topic in Germany that do not represent a consistent image of prevalence. The current article addresses prevalence and situational escalation factors as well as consequences of the incidents and wishes of the emergency medical services regarding attacks.

**Methods:**

Between May and August 2021, emergency medical services were surveyed using a mixed-methods approach, which included long-term data collection on the frequency of violent crime in the form of an online questionnaire and qualitative interviews of experts and victims.

**Findings:**

Verbal attacks in particular are part of the everyday working experience of emergency medical services. On average, 29% of respondents were insulted, harassed, or verbally threatened. Moreover, an average of 8% of those surveyed were also exposed to physical attacks. At the same time, the need for improvement regarding aftercare as well as education and training are expressed.

**Discussion:**

Education and training courses that raise awareness of dangers, consider de-escalation approaches, and address self-protection could reduce the risk of attacks and thus stress of this kind in everyday working life.

## Hintergrund

Nicht nur durch mediale Berichterstattung, sondern auch in politischen Debatten wird das Problem gewaltsamer Angriffe auf normdurchsetzende und helfende Funktionsträger*innen der Gesellschaft – wie auch auf Mitarbeiter*innen des Rettungsdienstes – in den Fokus gerückt. Ohne auf valide Angaben zurückgreifen zu können, basieren die Diskurse auf der Annahme, dass die Berufsgruppen zunehmend Gewalt ausgesetzt sind [1–3]. Neben verschiedenen Kampagnen^1^ zeigt sich anhand von Gesetzesänderungen das Engagement der Politik. Eine Novellierung der §§ 113 und 114 Strafgesetzbuch (StGB) im Jahr 2011 erweiterte den Anwendungsbereich von Widerstandsdelikten im Hinblick auf Rettungskräfte. § 113 StGB ist nun nicht mehr nur im Fall der Unterstützung von Diensthandlungen von Vollstreckungsbeamt*innen anwendbar, sondern eine Verurteilung kann auch im Fall der Behinderung oder des Angriffs bei Hilfeleistungen in Unglücksfällen bzw. in Not erfolgen. Ergänzend wurde der Strafrahmen von Widerstandshandlungen erhöht [4]. Eine erneute Änderung der genannten Paragrafen erfolgte im Jahr 2017 zum weiteren Schutz von Vollstreckungsbeamt*innen und Rettungskräften. Der neue § 114 StGB stellt den tätlichen Angriff nun gesondert unter Strafe [5]. Dafür wird in § 115 StGB die Anwendungsmöglichkeit auf den Rettungsdienst festgehalten, seit dem Jahr 2021 zählen zu dem dort umfassten Personenkreis auch Beschäftigte eines ärztlichen Notdienstes oder einer Notaufnahme [6].

Die Sinnhaftigkeit dieser gesetzlichen Änderungen bleibt jedoch umstritten [1–3]. So existierte keine sogenannte Strafbarkeitslücke, sondern Delikte, die nun nach §§ 113, 114 und 115 StGB strafbar sind, werden auch durch andere Straftatbestände (bspw. §§ 223, 224, 240 StGB) abgedeckt [1, 3]. Ähnlich sind auch die Strafrahmenerhöhungen in der Praxis nicht relevant, da die hier infrage kommenden Taten ohnehin auch andere Straftatbestände mit höheren Strafrahmen erfüllen. Dass ein wissenschaftlicher Nachweis einer generalpräventiven Wirkung von Strafschärfungen im Sinne einer Abschreckung fehlt, scheint dabei ebenfalls ignoriert worden zu sein [1–3]. Zudem sei die Erweiterung des Personenkreises auf Hilfeleistende bei Unglücksfällen und in besonderen Situationen bereits im Jahr 2011 mit dem eigentlich geschützten Rechtsgut des § 113 StGB nicht mehr vereinbar, bei welchem es sich nach herrschender Meinung nicht um die Person, sondern die Vollstreckungshandlung handelte [1, 3].

Insgesamt drängt sich die Vermutung auf, dass hier ein symbolischer Akt der Anerkennung bestimmter Professionen als herausragende Opfergruppe erfolgte, was auch der entsprechenden Gesetzesbegründung zu entnehmen ist [7]. Durch diese Loslösung von anderen Tatbeständen, die eigentlich gleichartige Sachverhalte abdecken, werden die Ehre und der Respekt gegenüber bestimmten Berufsgruppen gesondert geschützt und der Angriff darauf unter Strafe gestellt [8]. Dabei bleibt fraglich, ob die davon versprochene Rückenstärkung von Einsatzkräften tatsächlich auf diesem Weg erfolgen kann, denn eine faktische Senkung der Häufigkeit von derartigen Straftaten ist dadurch nicht zu erwarten [1–3].

**Figure 1:**
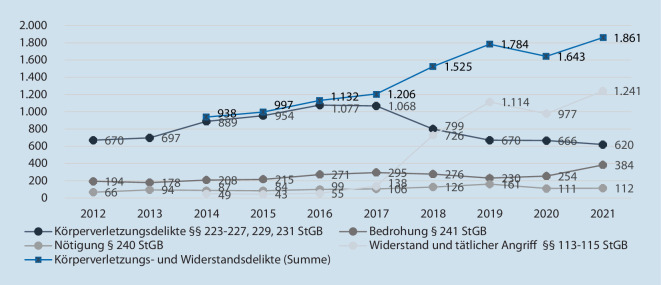

Die Grundlage der Gesetzesänderung war ein erkennbarer Anstieg entsprechender Delikte zum Nachteil von Rettungsdienstmitarbeiter*innen und weiteren Berufsgruppen in der Polizeilichen Kriminalstatistik (PKS). Diese erfasst die Opfergruppe seit 2011 unter der Bezeichnung „sonstige Rettungsdienste“ gemeinsam mit anderen Professionen, wie bspw. Mitarbeitenden des Technischen Hilfswerks, nicht jedoch der Feuerwehr. Abb. 1 gibt einen Überblick über einzelne polizeilich bekannt gewordene Delikte gegen Rettungsdienstpersonal in den letzten 10 Jahren.

Betrachtet man die Entwicklung dieser Zahlen, fällt bei Körperverletzungsdelikten ein Anstieg bis zum Jahr 2017 auf. Der darauffolgende abrupte Rückgang ist auf die erwähnte Erweiterung der Widerstandsdelikte zurückzuführen: Hier erfolgte ein Anstieg von 138 Delikten im Jahr 2017 auf 726 im Jahr 2018, gefolgt von einer weiteren Zunahme auf 1241 Fälle im Jahr 2021. Demnach müssen die Fälle im Gesamten bewertet werden, da seit 2017 eine andere rechtliche Würdigung gleichgelagerter Taten vorliegt. Die blaue Linie zeigt die summierten Fallzahlen. Hier wird ebenfalls der stetige Anstieg von polizeilich registrierten Angriffen auf Rettungsdienstpersonal deutlich.

Dabei ist zu bemerken, dass Angaben der PKS einigen Einschränkungen unterliegen, die es zu berücksichtigen gilt, um keine fehlerhaften Schlüsse zu ziehen. So kann ein Anstieg im Hellfeld – der polizeilich bekannt gewordenen Fälle – auch immer eine Zunahme der Anzeigebereitschaft abbilden. Eine solche ist in Zeiten, in denen einem Thema vermehrt Aufmerksamkeit zuteilwird, durchaus anzunehmen [3]. Ergänzend muss festgehalten werden, dass die PKS eine Eingangsstatistik ist und die strafrechtliche Einschätzung der Polizei abbildet; die staatsanwaltschaftliche und gerichtliche Bewertung wird aus ihr nicht ersichtlich [1].

Aus diesen Gründen ist eine Ergänzung der Daten durch weitere (Dunkelfeld‑)Befragungen sinnvoll, um einen Eindruck der tatsächlichen Prävalenzen von Angriffen gegen Einsatzkräfte zu erlangen. Bisher bilden die Grundlage der Erkenntnisse in der Regel Retrospektivbefragungen, in denen Häufigkeiten zu Angriffen im letzten Jahr oder im gesamten Berufsleben abgefragt werden [10–16].^2^ Die herangezogenen Prävalenzen bergen die methodische Schwierigkeit, dass die tatsächliche Anzahl der erlebten Angriffe über einen langen Zeitraum kaum überblickt werden kann. Verzerrungen aufgrund des Erinnerungsvermögens können dazu führen, dass außergewöhnliche oder schwerwiegende Ereignisse besonders herausstechen. Gleichzeitig können weniger schwerwiegende oder wiederkehrende Fälle – bspw. Beleidigungen – als Normalität gewertet werden und daher in Vergessenheit geraten [9]. Zudem ist davon auszugehen, dass die Art der Frageformulierung Einfluss auf das Antwortverhalten hat. Einige Studien verzichteten etwa auf die Vorgabe der Antwortkategorie „nie“, wodurch die Ergebnisse eine offensichtliche Beeinflussung erfahren dürften [10].

Die bestehenden Studien gehen von unterschiedlichen Gewaltbegriffen aus und umfassen verschiedene Grundgesamtheiten, wodurch die Vergleichbarkeit eingeschränkt ist [9]. Besteht eine Unterscheidung nach verbalen und körperlichen Angriffen, zeigte sich, dass Erstere sehr viel häufiger vorkommen als physische Gewalt. In einem Zeitraum von 12 Monaten wurde ein Anteil zwischen 38 % [11] und knapp 98 % [12] der Befragten identifiziert, der verbale Gewalt erleben musste. Bei körperlicher Gewalt lagen die Ergebnisse mit knapp 13 % [11] und 84 % [14] noch weiter auseinander.

Ziel der vorliegenden Untersuchung ist es, anhand einer methodischen Vorgehensweise, die nicht auf Erinnerungen basiert, sondern Vorfälle über einen längeren Zeitraum von Mai bis August 2021 zeitnah erfasste, die unklare Studienlage hinsichtlich Prävalenzen von Angriffen auf Rettungsdienstpersonal aufzuhellen. Ergänzend sollen situative Ansatzpunkte sowie Folgen der Taten und Wünsche der Einsatzkräfte in Bezug auf Angriffe dargelegt werden.

## Methoden

Um die Häufigkeiten von Angriffen gegen Mitarbeitende des Rettungsdienstes darzustellen und außerdem Angaben zu den Folgen wie auch Wünschen von den Bediensteten in diesem Kontext zu bekommen, wurden quantitative sowie qualitative Daten erhoben.

### Quantitative Erhebung

Den bereits dargestellten Problemen der bisherigen Studien zur rückwirkenden Ermittlung von Prävalenzen wurde in vorliegender Studie durch eine andere Vorgehensweise entgegengewirkt. Mit dem sogenannten Ereignisprotokoll wurden die Teilnehmenden gebeten, das Vorkommen aller erlebten Angriffe im beruflichen Kontext über einen Zeitraum von 4 Monaten in wöchentlichen Zeitintervallen zu protokollieren. Dabei war es ebenso wichtig, auch das Nichtvorkommen von Angriffen zu erheben.

Entsprechend wurde in 16 aufeinanderfolgenden Onlinekurzfragebögen das Vorkommen bzw. die Häufigkeit von verbalen und körperlichen Angriffen, Sachbeschädigung, Diebstahl und dem bewussten Behindern und Stören von Einsatzmaßnahmen abgefragt. Die 3 letztgenannten Kategorien, die vermeintlich weniger als direkter Angriff gegen Einsatzkräfte selbst gerichtet waren, wurden bewusst miteinbezogen, da auch solche Vorfälle den Einsatzablauf stören und auf Dauer zu Belastungen für die Beschäftigten führen können. Zudem sollten die Teilnehmenden die Möglichkeit haben, sämtliche Ereignisse vollständig zu dokumentieren. Die Antwortkategorien zur Häufigkeit waren „nie“, „einmal“, „zweimal“ bis hin zu „dreimal und häufiger“. Ebenso wurden Angaben wie Alter, Geschlecht, Berufserfahrung sowie die Einwohneranzahl des hauptsächlichen Einsatzgebietes erfasst.

Ergänzend zu diesem ersten Befragungsteil, der nur die Prävalenzen von Angriffen ermittelte, wurden in einem zweiten Teil detailliertere Informationen zu den Vorfällen abgefragt. Nur Personen, die einen Vorfall erlebt hatten, wurden um Angaben zu Merkmalen und Rahmenbedingungen der Situation, zu den Betroffenen und Angreifenden sowie den Folgen gebeten. Dabei handelte es sich mit einer Ausnahme, in der eine kurze Situationsbeschreibung mit eigenen Worten ersucht wurde, um geschlossene Fragen.

Um einen möglichst großen Rücklauf zu erlangen, wurde bundesweit auf das Forschungsprojekt aufmerksam gemacht. So wurden sämtliche großen Hilfsorganisationen mit dem Schwerpunkt Rettungsdienst auf Bundesebene kontaktiert und um eine Weiterleitung und Bekanntmachung der Studie gebeten. Hinzukommend wurden der Deutsche Feuerwehrverband und die Arbeitsgemeinschaft der Leiter der Berufsfeuerwehren in der Bundesrepublik Deutschland im Deutschen Städtetag auf die Befragung hingewiesen und gebeten, sie unter ihren Mitgliedern zu bewerben. Auch wenn bei derartigen Längsschnittstudien, die eine längerfristige Bereitschaft zur Mitwirkung erfordern, das Engagement geringer ausfällt, war die Beteiligung aus dem Bereich Rettungsdienst besonders zurückhaltend. Es konnte nicht nachvollzogen werden, inwiefern bereits die Weiterleitung gescheitert ist.

Die Befragungen erfolgten in Form eines Onlinefragebogens im Zeitraum von Mai bis August 2021. Nach der Datenbereinigung wurden quantitative Datenauswertungen mittels der Statistiksoftware SPSS (IBM, Armonk, NY, USA) ausgeführt. Insgesamt nahmen an der quantitativen Befragung 60 Personen aus dem Bereich Rettungsdienst und Notfallmedizin teil. Dabei handelte es sich zu 88 % um nichtärztliches Rettungsdienstpersonal. Über die Hälfte der Teilnehmenden war unter 30 Jahre alt und der überwiegende Anteil männlich (76 %). Nicht alle Teilnehmenden nahmen über den gesamten Befragungszeitraum von 16 Wochen teil, die wöchentlich schwankende Teilnehmendenzahl lag zwischen 17 und 36, im Durchschnitt bei 32 Personen.

### Qualitative Interviews

Im Rahmen der allgemeinen Bekanntmachung der Studie und Akquise für die Teilnahme an den Befragungen wurden zudem Interviewpartner*innen aus den genannten Personengruppen für qualitative Interviews gesucht, um weitere Details sowie Möglichkeiten zur Reduzierung der Häufigkeit von Angriffen zu ermitteln. Die Rückmeldungen darauf waren rar, sodass gezielte Nachfragen im Kreis der Verbund- und assoziierten Partner des Projektes erfolgen mussten. Die leitfadengestützten, problemzentrierten Interviews wurden zum einen mit Expert*innen in leitender Position mit Überblick über die Tätigkeit im Allgemeinen und die Gewaltthematik im Speziellen (*n* = 2) und zum anderen mit betroffenen Personen, die selbst einen verbalen und/oder körperlichen Angriff (in den letzten 2 Jahren) erlebt hatten (*n* = 2), geführt.

Der Leitfaden umfasste dabei die Themen Ansehen der Berufsgruppe, die Rolle von und die Gründe für Angriffe im Arbeitsalltag, der interne Umgang mit solchen Vorfällen und organisationale Präventionsmaßnahmen zur Verhinderung von Angriffen. Betroffene wurden zudem direkt zu dem Vorfall selbst, der Zusammenarbeit mit Kolleg*innen, der erhaltenen Nachbetreuung und ihren Wünschen hinsichtlich dieser Aspekte befragt.

Die wörtlich transkribierten Interviews wurden mithilfe der qualitativen Inhaltsanalyse unter Anwendung der Analysesoftware MAXQDA (VERBI – Software. Consult. Sozialforschung. GmbH, Berlin, Deutschland) ausgewertet. Zur Strukturierung und Reduzierung des Materials auf wesentliche Aussagen wurden Codes entwickelt, die auf die Interviews angewendet wurden.

## Ergebnisse

### Häufigkeiten von Angriffen und Störungen des Rettungsdienstes

**Figure 2:**
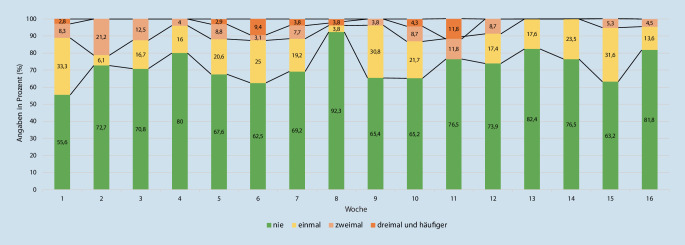

Bei Betrachtung der Häufigkeit von Angriffen wird deutlich, dass verbale Angriffe wie Beleidigungen oder Bedrohungen im Berufsalltag von Rettungsdienstpersonal regelmäßig erlebt wurden. Über den Gesamtzeitraum hinweg berichteten im Durchschnitt 29 % der Teilnehmenden mindestens einmal in der Woche von einem verbalen Angriff. In den meisten Fällen wurde dabei nicht mehr als ein Angriff rückgemeldet (19 %), insgesamt wiesen die wöchentlichen Angaben jedoch große Schwankungen auf (Abb. 2). Die durchschnittliche Häufigkeit von verbalen Angriffen sank im Verlauf der Befragung geringfügig: Während im ersten Monat 31 % der Befragten pro Woche mindestens einen verbalen Angriff erlebten, waren es im letzten Monat durchschnittlich 24 %.

**Figure 3:**
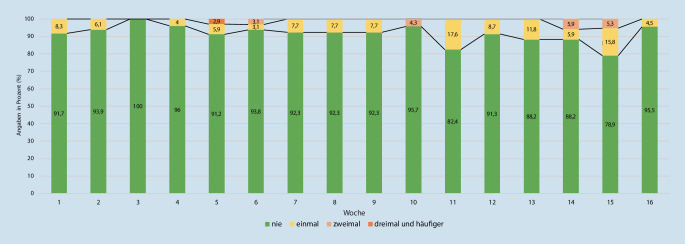

Im Mittel gaben pro Woche knapp 8 % der Befragten an, mindestens einen körperlichen Angriff erlebt zu haben. Körperliche Übergriffe wurden demnach deutlich seltener, aber dennoch regelmäßig berichtet und wiesen kaum Schwankungen während des Gesamtzeitraums auf (Abb. 3).

Von mindestens einer Sachbeschädigung in der abgefragten Woche berichteten rund 5 % der Befragten. Mindestens ein Diebstahl von Material oder Equipment wurde im wöchentlichen Durchschnitt von 1,5 % der Befragten angegeben. Eine oder mehr Störungen des Einsatzablaufs erlebten pro Woche durchschnittlich 22 % der Befragten.

Hinweise darauf, wie und wann sich die Situationen abspielten, konnten durch den zweiten Abschnitt der Befragung ermittelt werden, bei dem Betroffene nach detaillierteren Informationen zu den Vorfällen gefragt wurden. Es wurden im gesamten Erhebungszeitraum 66 Angriffssituationen gegenüber Rettungsdienstpersonal genauer dargelegt.

Bei verbalen Angriffen wurden Beleidigungen und Beschimpfungen am häufigsten genannt (Tab. 1). Ebenfalls oft berichteten die Befragten von verbalen Bedrohungen oder dem Androhen körperlicher Gewalt durch Gestik und Körperhaltung des Gegenübers. Bei körperlichen Angriffen überwogen Tritte und Schläge sowie Schubsen und Stoßen, Festhalten und Anspucken (Tab. 2).

**Table 1:** 

Arten verbaler Angriffe	*n*	%
Beleidigung oder Beschimpfung	35	29,2
Verbale Bedrohung	34	28,3
Androhung von körperlicher Gewalt durch Gestik und Körperhaltung	21	17,5
Nötigung oder Erzwingen eines bestimmten Verhaltens	17	14,2
Sexualisierte Beleidigungen oder Beschimpfung	6	5,0
Rassistische Beleidigungen oder Beschimpfung	3	2,5
Bedrohung mit Gegenständen	2	1,7
Bedrohung mit Waffen	2	1,7

**Table 2:** 

Körperlicher Angriff	*n*	%
Schlagen oder Treten	15	31,9
Schubsen oder Stoßen	10	21,3
Festhalten oder Anpacken	8	17,0
Anspucken	6	12,8
Verteilung weiterer Körperflüssigkeiten	3	6,4
Beißen oder Kratzen	3	6,4
Haare ziehen	1	2,1
Angriff mit Gegenstand (z. B. mit etwas beworfen werden)	1	2,1

Von den 66 ausgefüllten ausführlichen Ereignisprotokollen wurden 14 Erlebnisse als bedrohlich oder sehr bedrohlich bewertet. Von diesen als besonders schwerwiegend eingeschätzten Vorfällen waren die Betroffenen in nahezu allen Fällen (*n* = 11) verbaler Gewalt und bei etwas mehr als der Hälfte der Fälle (*n* = 8) körperlicher Gewalt ausgesetzt. Als besonders schwerwiegend wurde hierbei die verbale Bedrohung und das Androhen von Gewalt durch Gestik und Körperhaltung sowie Schlagen und Stoßen empfunden.

Bezogen auf die Gesamtheit der Angriffe traf das Rettungsdienstpersonal bei der Mehrzahl der Vorfälle als Erstes vor Ort ein (71 %). Eher selten waren schon Einsatzkräfte der Polizei (18 %) oder eigene Kolleg*innen (9 %) vorher an der Einsatzstelle.

Die Hälfte der Angriffe wurde durch die behandelten Patient*innen selbst verübt. Bei den übrigen angreifenden Personen handelte es sich um Angehörige oder Bekannte dieser Personen (32 %) und seltener um unbeteiligte Personen (17 %). Der Großteil der Angreifenden war männlich (76 %) und mittleren Alters (30–50 Jahre; 45 %). Bei rund 2 Drittel der Vorkommnisse war die angreifende Person nach Einschätzung der Befragten alkoholisiert (67 %), bei beinahe jedem dritten Angriff stand sie unter anderen Drogen (29 %). Ein Drittel der angreifenden Personen wurde von den Betroffenen selbst als psychisch auffällig beschrieben. Nur bei 12 % der Angreifenden wurde keines dieser Merkmale genannt.

Hervorzuheben ist, dass über die Hälfte (58 %) der Einsatzkräfte die Angriffe als überraschend und unerwartet beschrieben. Knapp ein Drittel berichtete von einer gewissen Vorahnung aufgrund einer bereits angespannten Lage (29 %). Mit einer konkreten Gefahrensituation rechneten nur 14 % der Befragten, bspw. durch die Einsatzmeldung.

Der Zeitpunkt, zu dem sich die Angriffe ereigneten, war größtenteils während der Anamnese und Diagnostik (62 %). Dass die Angriffe direkt zu Beginn beim Eintreffen an der Einsatzstelle (14 %) oder beim anschließenden Transport (15 %) verübt wurden, traf jeweils seltener als in jedem sechsten Fall zu. Nur in Ausnahmefällen ereignete sich der Vorfall bei der Übergabe an weiterbehandelnde Dritte (4 %).

### Folgen von Angriffen

In den Interviews wurden die beiden rekrutierten Gesprächspartner*innen konkret nach den Folgen und Konsequenzen solcher Angriffe gefragt. Es kann positiv hervorgehoben werden, dass selten von langwierigen und schwerwiegenden körperlichen Beeinträchtigungen berichtet wurde. Eher waren jedoch psychische Belastungen von Bedeutung, die die Betroffenen – teilweise auch über einen längeren Zeitraum – beeinträchtigten. Dazu gehörten Flashbacks, die in Situationen hervorgerufen wurden, die dem Setting des Vorfalls ähnelten. Ebenfalls wurde von einem veränderten Verhalten im Berufsalltag, bspw. in Form von Vermeidungsstrategien oder dem frühzeitigeren Hinzuziehen der Polizei berichtet. Aber auch beim Einsatzgeschehen selbst wurden Verhaltensänderungen geschildert, die sich insbesondere in einem gesteigerten Gefahrenbewusstsein, einer höheren Sensibilisierung für mögliche Eskalationsfaktoren und einem höheren Fokus auf Eigenschutz äußerten. Auffallend ist dabei, dass Betroffene nach einem Angriff teilweise eigeninitiativ und in ihrer Freizeit an Schulungen zur Selbstverteidigung teilnahmen, um ihr Sicherheitsempfinden zu steigern und um zukünftige gewalttätige Ereignisse abwenden zu können.

Zusätzlich zu den Folgen, die den Arbeitsalltag betrafen, ergaben sich auch Auswirkungen auf das Privatleben der Betroffenen. Es wurden Schlafstörungen oder auch Angstzustände geschildert wie auch ein wiederkehrendes intensives Nachdenken über den Vorfall.

Die justizielle Aufarbeitung wurde ebenfalls als belastend wahrgenommen. Allerdings wurde sie von den Betroffenen auch positiv als Anerkennung des Vorfalls wahrgenommen und ihnen dadurch eine Möglichkeit gegeben, mit dem Fall abzuschließen.

### Nachbetreuung nach Angriffen

Die Interviews ergaben, dass Nachbesprechungen nach erlebten Angriffen meist nur auf kollegialer Ebene und im informellen Rahmen stattfanden. Hier vermissten die Interviewpartner*innen speziell vonseiten der Vorgesetzten eine deutliche Wahrnehmung und Anerkennung der durch das Erlebte entstandenen Belastungen. Vor allem hinsichtlich verbaler Angriffe bestehe aktuell kaum Bewusstsein über mögliche Folgen für die Betroffenen. Zudem wurden eine höhere Akzeptanz und Sensibilisierung für eine adäquate Nachbetreuung, bspw. in Form von psychologischer Begleitung gewünscht. Wenn bereits derartige Angebote vorhanden waren, wurde bemängelt, dass nicht ausreichend darauf aufmerksam gemacht wurde und es im Arbeitsalltag kaum Wissen über diese Möglichkeiten gäbe. So wurde von den Vorgesetzten angenommen, dass die Beschäftigten über diese Angebote Bescheid wüssten und auf eine informelle Verbreitung dieser Informationen gesetzt.

### Ausbildungen und Schulungen mit Bezug zu Angriffen

Gerade in der Ausbildung sowie in Schulungen und Fortbildungen wurde nach Einschätzung der Interviewpartner*innen die Gewaltthematik noch nicht hinreichend behandelt. Letztere sollten regelmäßig stattfinden, um die Inhalte immer wieder ins Bewusstsein der Rettungsdienstmitarbeitenden zu rufen. Dabei sollten sich die Schwerpunkte den Interviewpartner*innen zufolge auf verschiedene Bereiche konzentrieren.

Nötig wäre … die weitere Vertiefung dieser Geschichten in der Ausbildung zum Rettungssanitäter. Das heißt die Gefahrenlehre, die Eigensicherung und dann die Deeskalation und dann den Umgang mit gewalttätigen Situationen [Expert*in_1].

So wurde das Gefahrenbewusstsein der Beschäftigten im Rettungsdienst als nicht ausreichend angesehen. Dies treffe insbesondere auf jüngere Mitarbeitende zu, die nicht auf eine umfangreiche Berufserfahrung zurückgreifen und daraus ableitend Situationen hinsichtlich ihres Gefahrenpotenzials schlechter einschätzen könnten. Eine intensivere Einbindung bereits in der Ausbildung könnte dazu beitragen, kritische Situationen frühzeitig richtig einzuschätzen und gegebenenfalls die eigene Strategie zu ändern, bspw. in Form von Rückzug oder Unterstützungsanforderung der Polizei.

Ich glaube tatsächlich eher, dass man das Sicherheitsbewusstsein schärfen muss bei den Rettungsdienstmitarbeitern. Sie müssen viel früher, viel genauer, viel gezielter erkennen können, woraus könnte eine Gefahrensituation entstehen [Expert*in_2].

Weiterer Schulungsbedarf wurde im Bereich der Deeskalation gesehen. Hiervon erhofften sich die Befragten, in kritischen Situationen adäquater handeln zu können und damit im Idealfall eine Eskalation abzuwenden. Aktuell bestehen nach Einschätzung der Befragten bei Rettungsdienstmitarbeitenden wenige derartige Kenntnisse und Inhalte würden nur vereinzelt vermittelt. Stattdessen wurde eine Verankerung von Deeskalationsmaßnahmen im festen Ausbildungskanon des Rettungsdienstes gefordert, um Beschäftigten deeskalierende Handlungs- und Kommunikationsweisen mitzugeben, die in potenziell bedrohlichen Situationen schnell abgerufen werden können. Dazu gehörten u. a. die Wissensvermittlung über die Auswirkungen des eigenen Auftretens, die Verwendung einfacher Sprache gegenüber Patient*innen, aber auch die Bereitstellung vorformulierter Sätze.

Manche Leute sind wie gesagt rhetorisch vielleicht dazu veranlagt, die können das und manche können es halt nicht und man *darf* diese Menschen nicht alleine lassen, ja. Man muss da Möglichkeiten vermitteln, vielleicht auch standardisierte, vielleicht auch fest fixierte Sätze … [Expert*in_2].

Hierfür wurde eine Aneignung nicht nur auf theoretischer Ebene, sondern auch in praktischen Übungen und realitätsnahen Schulungen angeregt.

Um auf Situationen vorbereitet zu sein, die nicht mehr deeskaliert werden können und in denen es tatsächlich zu einer Auseinandersetzung kommt, müssten laut Gesprächspartner*innen vor allem Selbstverteidigungsstrategien vermittelt werden. Beispielhaft wurde eine verletzungsfreie Abwehr von Angriffen oder ein erfolgreicher Rückzug aus kritischen Situationen genannt. Daran anknüpfend wurde Aufklärung über die adäquate Nutzung von Notrufvorrichtungen gewünscht. Denn obwohl Notrufmeldesysteme wie ein Notfallknopf vorhanden seien, würden diese auch in gefährlichen Situationen häufig nicht verwendet, da nicht ausreichend über deren Verwendung unterrichtet wurde und die Benutzung eher tabuisiert werde. Weiterhin wurden effiziente und schnelle Notfallverriegelungen der Einsatzwagen gefordert, um die Fahrzeuge als sicheren Rückzugsort bei physischen Übergriffen nutzen zu können.

Hinsichtlich des Austauschs mit anderen Einsatzkräften, speziell der Polizei, wurden besonders häufig gemeinsame berufsübergreifende Trainings und Schulungen gewünscht. Die Gesprächspartner*innen gaben an, dass es im Einsatzgeschehen gelegentlich zu Problemen in der Abstimmung mit anderen Einsatzkräften komme. Sie erhofften sich aus gemeinsamen Übungen, insbesondere in realitätsnahen Einsatzszenarien, ein besseres gegenseitiges Verständnis für die jeweiligen Bedürfnisse der anderen Berufsgruppen.

Also es gibt oftmals kein Verständnis für die Tätigkeit des anderen. Auch auf unserer Seite, ja. Ein Polizist hat natürlich an ’ne Einsatzstelle ganz, ganz andere Ansprüche wie wir zum Beispiel [Expert*in_2].

## Diskussion

Die Ergebnisse dieser Studie zeigen, dass verbale Angriffe wie Beleidigungen oder Bedrohungen im Berufsalltag von Rettungsdienstpersonal regelmäßig vorkommen. Der Anteil von Personen, die einen verbalen Angriff erlebten, schwankte in den Wochen von etwa einem Fünftel bis zu einem knappen Drittel. Auch wenn derartige Schwankungen bei der vorliegenden Fallzahl nicht überinterpretiert werden dürfen, könnte die vermehrte Anzahl an Beleidigungen und verbalen Angriffen zu Beginn der Befragung möglicherweise durch den zu diesem Zeitpunkt noch wirksamen Lockdown aufgrund der COVID-19-Pandemie erklärt werden, der zum einen für Unzufriedenheit in der Bevölkerung gesorgt hat und zum anderen zusätzliche Belastungen, bspw. durch Hygienemaßnahmen oder Personalausfälle, für Rettungskräfte darstellte. Ähnlich häufig wie Beleidigungen oder Bedrohungen wurde das Behindern von Einsatzmaßnahmen berichtet.

Bei körperlichen Angriffen, Sachbeschädigung und Diebstahl handelte es sich eher um Einzelfälle; die Anteile waren im einstelligen Prozentbereich. Das heißt dennoch, dass durchschnittlich etwa jede 12. Rettungskraft einen körperlichen Angriff pro Woche meldete.

Da andere Studien unterschiedliche Prävalenzzeiträume haben, ist ein Vergleich schwierig. Die Regelmäßigkeit von verbaler Gewalt gegenüber Mitarbeiter*innen des Rettungsdienstes, die sich auch in den meisten vorherigen Studien zeigte, konnte hier nicht nur bestätigt, sondern auch differenzierter ermittelt werden. Die unterschiedlichen Ergebnisse zu der Häufigkeit körperlicher Angriffe ergänzen hiesige Daten und unterstützen die Befunde, dass sie im Vergleich sehr viel seltener sind. Eine wichtige Erkenntnis, der es in der Praxis zu begegnen gilt, zeigt sich durch die Angaben zu der wahrgenommenen Bedrohlichkeit der Angriffe, die verdeutlichen, dass auch verbale Angriffe für die Mitarbeitenden des Rettungsdiensts eine enorme Belastung darstellen können.

Betrachtet man die konkreten Situationen, in denen es zu Angriffen kommt, ließ sich feststellen, dass der Umgang mit berauschten oder psychisch auffälligen Personen ein besonderes Gefahrenpotenzial darstellt. Bei Betrachtung des Zeitpunktes der Angriffe während des Einsatzes ergab sich, dass bei dem überwiegenden Teil der Angriffe vorab höchstwahrscheinlich eine gewisse Interaktion zwischen Patient*in und Einsatzkraft stattgefunden hat. Eventuelle Warnsignale hätten somit wahrgenommen werden können.

Bezüglich der Angriffsfolgen wird deutlich, dass diese sowohl im beruflichen Kontext als auch im privaten Bereich auftreten können. Eine besondere Rolle für die Betroffenen spielt insbesondere die Anerkennung der Belastungen des Erlebten, die bei den Vorgesetzten teilweise vermisst wird. Insgesamt bestehen im Hinblick auf die Nachbetreuung nach einem Angriff bisher in vielen Fällen – insbesondere bei verbalen Angriffen – keine festen Strukturen und Abläufe innerhalb der einzelnen Organisationen. Die besondere Bedeutung der Anerkennung der widerfahrenen Gewalt als Unrecht und gesellschaftlich intolerabel zeigte sich auch im Zusammenhang mit der justiziellen Aufarbeitung, die den Betroffenen eine gewisse Befriedung verschafft.

Nach Ansicht der Interviewpartner*innen sollte die Gewaltthematik in der Ausbildung sowie in Schulungen und Fortbildungen ausführlicher behandelt werden, denn bei Angriffen, die sich erst im Verlauf eines Einsatzes entwickelten, deuten die Ergebnisse der vorliegenden Studie darauf hin, dass Gefahrensignale möglicherweise zu spät erkannt wurden oder die Konfliktsituationen immer weiter eskalierten und schließlich in einem Angriff endeten. Den quantitativen als auch qualitativen Erkenntnissen zufolge könnten durch eine Gefahrensensibilisierung der Betroffenen Angriffe eventuell abgewendet werden. Schulungen zur mentalen Einstellung gegenüber Angriffen könnten zudem zu einer verstärkten Wachsamkeit an der Einsatzstelle beitragen, sodass bspw. ein Rückzug aus gefährlichen Situationen in zukünftigen Situationen möglicherweise noch rechtzeitig in Erwägung gezogen und umgesetzt werden könnte.

Vorliegende Studie unterliegt Grenzen in der Aussagekraft, die insbesondere auf die Stichprobengröße zurückzuführen sind. Trotz umfangreicher Bemühungen konnte nur eine relativ geringe Anzahl von Teilnehmenden akquiriert werden. Zur genaueren Betrachtung von Unterschieden von Angriffen hinsichtlich bestimmter Aspekte, wie bspw. Dem Alter der Mitarbeitenden oder zwischen Stadt und Land, wäre eine größere Beteiligung notwendig gewesen. Dennoch können die dargestellten Ergebnisse der innovativen Erhebungsmethode im Kontext bereits bestehender Erhebungen einen wichtigen Beitrag zur Aufklärung über die Thematik leisten.

## Fazit

Um Angriffen gegen Rettungskräfte angemessen zu begegnen und diese potenziell zu vermeiden, ist es wichtig, sie in den Fokus zu nehmen. Dies gilt für die Politik, die Rettungskräften die Wertschätzung entgegenbringen muss, die ihnen zusteht. Zudem muss die Wissenschaft bestehende Kenntnisse zu dem Phänomen erweitern und Präventionsansätze finden. Letztendlich gilt es, durch Aus- und Fortbildung Mitarbeitende des Rettungsdienstes für das Thema zu sensibilisieren, das bestehende Wissen zu vermitteln sowie Handlungsoptionen zur Vermeidung an die Hand zu geben.

## Footnotes

1 Bspw. „Polizei und Rettungskräfte für ein sicheres Deutschland“ des Bundesministeriums des Inneren und für Heimat (https://sicherheit.bund.de); „Respekt?! Ja bitte!“ der Deutschen Feuerwehr-Gewerkschaft und der Gewerkschaft der Polizei Nordrhein-Westfalen (https://respekt-ja-bitte.de).

2 Für einen Überblick über bestehende Studien siehe den Artikel von Rau und Leuschner [9].
